# Monodisperse upconversion GdF_3_:Yb, Er rhombi by microwave-assisted synthesis

**DOI:** 10.1186/1556-276X-6-267

**Published:** 2011-03-29

**Authors:** Haiqiao Wang, Thomas Nann

**Affiliations:** 1 Institute Materials for Electronics and Energy Technology (I-MEET), Friedrich Alexander Universität Erlangen-Nürnberg, Marternsstraße 7, 91058, Erlangen, Germany; 2 Ian Wark Research Institute, University of South Australia, Mawson Lakes, South Australia 5095, Australia

## Abstract

We have synthesized a variety of monodisperse colloidal GdF_3_:Yb, Er upconversion nanocrystals with different shape, size, and dopants by microwave-assisted synthesis. Typical upconversion emission from Er^3+ ^was observed. In addition to highly monodisperse spherical particles, we were able to prepare monodispersed rhombic-shaped slices that showed a tendency for self-assembly into stacks.

## Introduction

In recent publications we have shown that microwave-assisted synthesis allows for the preparation of highly monodisperse, spherical upconversion nanocrystals [1], as well as nanocrystals with unusual morphologies [2]. In this research letter, we report on the microwave-assisted synthesis of monodispersed spherical and rhombic GdF_3_-based nanoparticles, which show a high tendency for self-assembly in one- and two-dimensional superstructures.

Research on upconversion nanocrystals increased exponentially over the past several years (e.g., in 2000, one article on upconversion nanomaterials was published, in 2009, it have been 57) as the extremely attractive prospects for applications of these materials in bioanalytics [3], (cancer) therapy [4], and electro-optics [5]. And subsequently, it is concluded that the most efficient infrared-to-visible upconversion phosphors are Yb/Er or Yb/Tm co-doped fluorides, such as hexagonal phase NaYF_4 _[6,7], LaF_3 _[8,9], and orthorhombic phase YF_3 _[10], GdF_3 _[11]. Especially in the past few years, the NaYF_4_-based phosphors, as the highest efficient upconversion phosphors, with different morphologies and different dopants have been widely investigated, based on various synthesis procedures. However, GdF_3 _as one of the efficient upconversion phosphor host [8], not too much work has been reported focusing on Yb/Er codoped fluorecence upconversion. Although Tm, Dy, Ho, and Yb/Tm doped GdF_3 _has been reported [11-14]. As far as we know, in 1971, the preparation of GdF_3_:Yb, Er phosphor was reported firstly by Major et al. In their procedure, the oxide precursors were dissolved in high purity nitric acid and precipitated with excess hydrofluoric acid, and finally experienced calcination. They found that the color of the anti-Stokes luminescence of the Yb/Er doped GdF_3 _phosphors was controllable by preparation processes, and was associated with the crystal structure of the host lattices. And they gave a dominant green emission when excited by 940 nm infrared light [10]. In 2006, Fan et al. employed a hydrothermal synthesis procedure to produced Yb/Er codoped GdF_3 _nanoparticles. For their prepared sample, typical upconversion emission was observed but with much weaker intensity than that of bulk crystal [15].

Microwave-assisted synthesis of nanomaterials offers several interesting synthetic opportunities which are based on the specific microwave effects: (i) microwave irradiation is absorbed by polar and ionic substances only (dielectric heating); (ii) enhanced reaction rates can be observed; (iii) heterogeneous heating (viz. "hot spots") and wall effects can be suppressed [16]. Details on the microwave-assisted synthesis of AYF_4 _(A = Na, Li) nanocrystals can be found elsewhere [1]. This research letter is concerned with the effects of microwave irradiation on the shape evolution of GdF_3 _nanocrystals.

In this paper, we firstly presented the preparation of colloidal upconversion GdF_3_:Yb, Er nanocrystals, based on a new microwave-assisted synthesis method. We have synthesized a variety of GdF_3_-based upconversion nanocrystals with different shape, size, and dopants by microwave-assisted synthesis. In addition to highly monodisperse spherical particles, we were able to prepare monodisperse rhombic-shaped slices that showed a tendency for self-assembly into stacks.

## Experimental

According to our previous work [1], introduction of Li^+ ^can help to enhance the upconversion efficiency. So here in the procedure, Li^+ ^was used as well. In a typical synthesis (standard conditions), 0.115 mmol (13.8 mg) lithium trifluoroacetate (TFA), 0.083 mmol (41.2 mg) gadolinium TFA, 17 μmol (8.5 mg) ytterbium TFA, and 1.7 μmol (0.86 mg) erbium TFA were dissolved in 6 ml of a 1:1 (v:v) mixture of oleic acid and octadecene in a nitrogen atmosphere. The solution was thoroughly degassed at 120°C and transferred into a microwave reaction vessel. Then, the mixture was heated for 10 min at 290°C by microwave irradiation. The resulting nanocrystals were precipitated by addition of 3 ml of ethanol to the cold reaction solution and subsequent centrifugation. The supernatant was discarded and the nanocrystals were repeatedly washed with ethanol. Eventually, the particles were re-dissolved in chloroform or toluene for further studies.

Transmission electron micrographs (TEMs) were recorded on a JEOL 2000EX and a JEOL 2010 microscope at 200 kV. Selected area electron diffraction (SAED) patterns were measured on the same instrument. The X-ray diffraction (XRD) patterns were measured with a thermo ARL XTRA equipped with a Cu X-ray tube (λ = 1.5418 Å). Upconversion spectra were recorded on an Oceanoptics USB spectrometer and a home-made cuvette holder and excitation source (980 nm, 100 mW laser diode).

## Results and discussion

**Figure 1:**
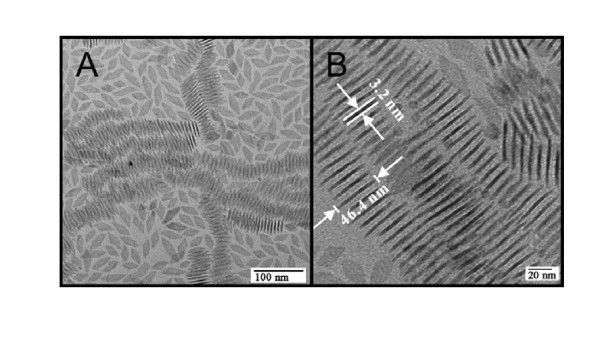
**TEMs of GdF_3 _nanocrystals**. **(A) **Overview graph of single nanocrystals and "stacks". **(B) **Blow-up of self-assembled nanocrystals.

Figure 1 shows TEMs of the resulting nanocrystals. It was observed that the particles were regularly shaped rhombic plates with approximately 3.2 nm thickness, approximately 45 nm in length and with a roughly calculated aspect ratio of 1:3. Both micrographs show clearly that the nanoplates have a strong tendency to form two-dimensional aggregates. This effect can be attributed to the minimization in energy, which is achieved by hydrophobic interaction of the surface ligands of the nanoparticles at their largest faces.

**Figure 2:**
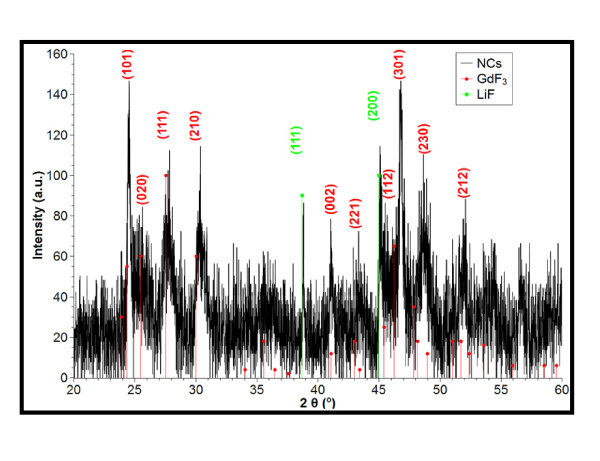
**XRD pattern of GdF3 nanocrystals as depicted in Figure 1**. Full circle (red): diffraction pattern according to JCPDS-file 012-0788; full square (green): diffraction pattern according to JCPDS-file 045-1460.

**Figure 3:**
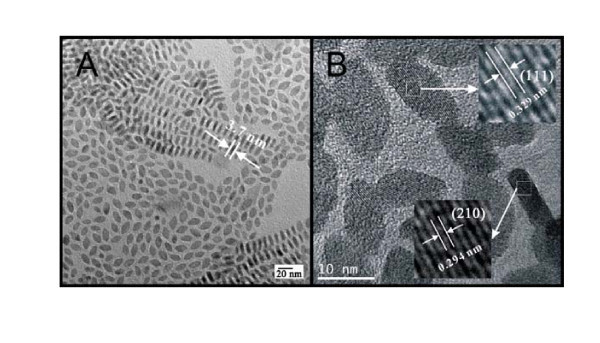
**TEMs of GdF3 nanocrystals prepared with 25% of the original concentration of reactants**. **(A) **Overview picture. **(B) **High-resolution TEM of single nanocrystals.

The XRD pattern of these particles (Figure 2) which shows orthorhombic phase GdF_3 _was obtained. Almost all the diffraction peaks of the XRD pattern can be assigned, respectively, to the planes of orthorhombic GdF_3 _crystalline (JCPDS-file 012-0788), as indicated (101), (020), (111), (210), (002), (221), (112), (301), (230), (212) in Figure 2 (red, round dot). However, two more weak diffraction peaks can also be observed at 2θ = 38.7° and 45°, which cannot be assigned to GdF_3_. We considered that these two weak peaks arose from the diffraction of LiF (JCPDS-file 045-1460) [17]. And it suggests that the Li^+ ^was not only introduced into the expected phosphor GdF_3 _crystal to replace some Gd^3+ ^sites as impurity but also a few LiF was formed. A further confirmation of a predominant GdF_3 _lattice can be found by measuring distances of the lattice fringes in the high resolution transmission electron microscopy (HRTEM). Lattice fringes were found with distances of 3.29 and 2.94 Å (cf. Figure 3B), corresponding well to the theoretically calculated distance of the {111} and {210} planes of the orthorhombic-YF_3 _space group GdF_3 _(orthorhombic phase JCPDS-file 012-0788), respectively. It is noteworthy that the same XRD patterns were observed for decreased and increased reactant concentrations.

In our previous work, we found that changing the reaction parameters (especially the concentration of reactants) in the synthesis of NaYF_4_-based upconversion nanocrystals, has crucial influence on the morphology of the resulting particles [2]. In this work, we observed that, based on our precursors, GdF_3 _nanocrystals were obtained and always adopt a rhombic shape under microwave irradiation, even when other synthesis parameters were changed.

Figure 3 shows TEMs of GdF_3 _nanocrystals that were synthesized under the above conditions, but with 25% of the original concentration of reactants. The particles are smaller than the ones displayed in Figure 1 (approximately 15-18 nm in length), but have roughly the same thickness and aspect ratio. Therefore, the rate of growth along the "edges" of the particles has to be much faster as compared to the primary faces ({111} and {210}). Figure 3B shows a HRTEM of the same particles. It can be observed that the lattice fringes are always aligned with one edge of the rhombi. This observation and the overall shape of the nanocrystals are in agreement with the anticipated orthorhombic-YF_3 _space group [18].

**Figure 4:**
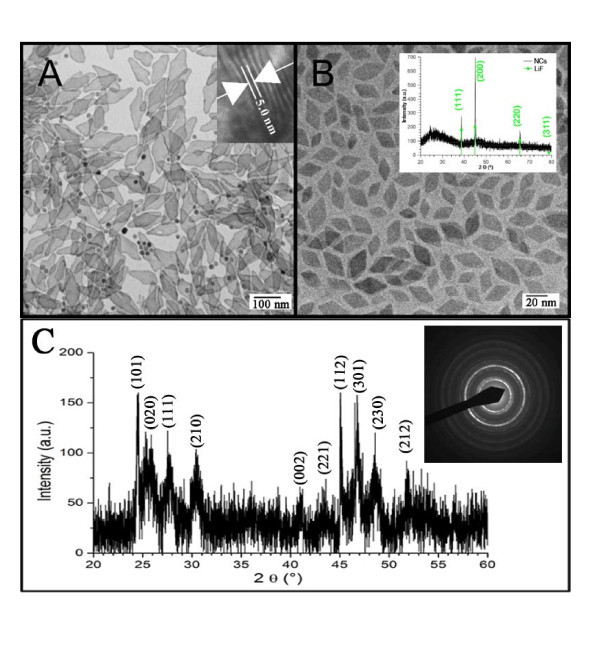
**TEM, XRD and SAED characterization of obtained samples**. **(A) **TEM micrograph of GdF_3 _nanocrystals synthesized at five times the standard concentration. **(B) **LiF nanocrystals synthesized by traditional conductive heating at the same conditions like A. Inset: XRD pattern of the obtained nanocrystals. **(C) **XRD pattern of GdF_3 _nanocrystals synthesized at five times the standard concentration. Inset: SAED pattern of the prepared sample.

When the concentration of reactants was increased by a factor of five, compared with the standard conditions, the nanocrystals were still predominantly rhombic in shape (in addition, some spherical particles were observed), but approximately 150 nm in length and 5 nm in thickness (cf. Figure 4A). This finding confirms further that the nanocrystals grow preferentially in two dimensions. Again, the XRD pattern is associated well with the orthorhombic phase GdF_3_, and without diffraction peaks of LiF any more (Figure 4C). Figure 4C inset shows the SAED pattern of this prepared sample. Figure 4B shows the TEM of the obtained nanocrystals that were prepared under identical conditions as the ones in Figure 4A, but using traditional conductive heating. Shape and size of these particles are roughly in the order of magnitude of the standard conditions described above. However, the XRD pattern (inset Figure 4B) of these nanocrystals shows that mainly cubic LiF nanocrystals have been synthesized.

**Figure 5:**
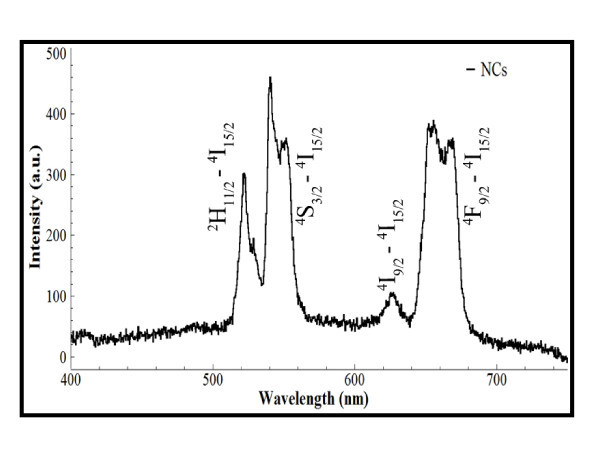
**Upconversion spectrum of GdF_3_:Yb, Er nanocrystals under excitation of 980 nm**.

Figure 5 shows the upconversion emission spectrum of the nanocrystals from Figure 4A, under 980 nm near infra-red (NIR) excitation. Mainly two emission bands were observed, with emission peaks at 521, 545, and 660 nm. These emission peaks can be attributed to the 4f-4f transitions of the Er^3+ ^ions. The green emission accounts for the ^2^H_(11/2)_, ^4^S_(3/2) _→ ^4^I_(15/2) _transition, the red emission is caused by the ^4^F_(9/2) _→ ^4^I_(15/2) _transition. The difference compared with typical reported Yb/Er emission spectrum is that a weak red emission at approximately 628 nm was observed, which could be attributed to the transition ^4^I_(9/2) _→ ^4^I_(15/2)_. Based on Yb/Er codopants, the observed upconversion efficiency of the GdF_3_-based nanocrystals is relatively lower than that of the NaYF_4_-based nanocrystals in our lab [1]. Comparing these values with data from the literature shows that the microwave-assisted synthesis does not influence the optical properties of the nanocrystals *per se*.

**Figure 6:**
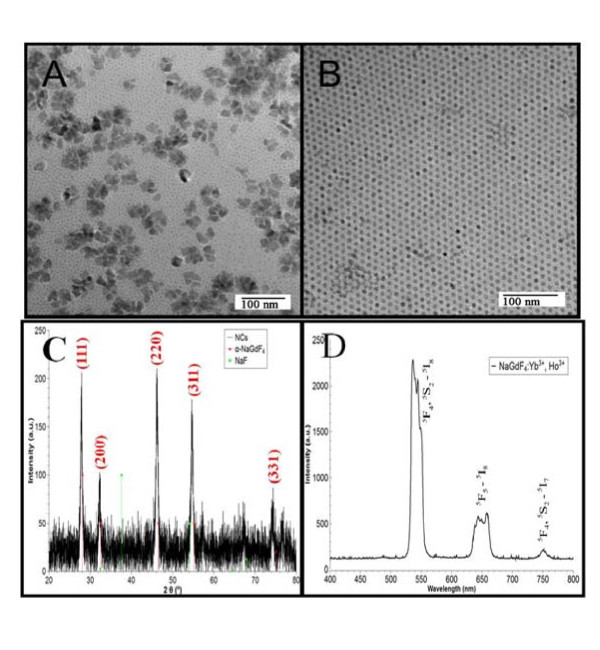
**TEMs of NaGdF4:Yb, Ho nanocrystals**. **(A) **Five times standard concentration; **(B) **25% standard concentration; **(C) **XRD pattern of NaGdF_4_:Yb, Ho nanocrystals; **(D) **Upconversion spectrum of NaGdF_4_:Yb, Ho nanocrystals under excitation of 980 nm.

In order to establish the reason for the strict rhombic shape of the nanocrystals, we replaced lithium with sodium in the above synthesis method. Yb/Ho codoped NaGdF_4 _nanocrystals were synthesized under the same conditions as above. Figure 6A shows the TEMs of the resulting particles with high and low reactant concentration, respectively. It can be seen that monodisperse, spherical particles were synthesized at low reactant concentration, and a bimodal distribution of monodisperse, spherical, and irregular larger nanocrystals at higher concentrations. Hence, these particles adopted a completely different morphology compared with GdF_3_. Figure 6C shows a XRD pattern of these particles, which confirms that the nanocrystals have crystallized in the cubic α-NaGdF_4 _phase (JCPDS 27-697). Therefore, we can conclude that the rhombic morphology of the GdF_3 _nanocrystals was primarily driven by the crystal lattice. Under the 980 nm excitation, mainly three emission bands were observed (Figure 6D). Predominantly green upconversion luminescence was observed at 542 nm, corresponding to the transition from the ^5^F_4 _and ^5^S_2 _to the ^5^I_8 _ground state. A weaker red and NIR upconversion luminescence was observed at 650 and 751 nm, which could be attributed to the transition from the ^5^F_5 _→ ^5^I_8 _and ^5^F_4_, ^5^S_2 _→ ^5^I_7 _states, respectively, which is in agreement with data from the literature.

## Conclusions

Our experimental results allow for three major conclusions:

1. The presence of lithium does not impair the predominant orthorhombic-YF_3 _space group of GdF_3 _at all concentrations tested.

2. The crystallization in the orthorhombic-YF_3 _space group and the rhombic shape of the nanoparticles are specific microwave effects. Conductive heating leads to completely different nanocrystals, although rhombic in shape.

3. The optical properties (viz. upconversion) of the nanocrystals seem to be unaffected by the microwave-assisted synthesis method.

Thus, beyond being rapid and easy to use, microwave-assisted synthesis of upconversion nanocrystals allows for the crystallization of new nanocrystals and morphologies. Rhombic plates, like the ones synthesized in our study, might be key to self-assembly or supramolecular strategies towards an improvement of the upconversion quantum yield.

## Abbreviations

SAED: selected area electron diffraction; TFA: trifluoroacetate; TEM: transmission electron microscopy; XRD: X-ray diffraction; HRTEM: high resolution transmission electron microscopy; NIR: near infra-red.

## Competing interests

The authors declare that they have no competing interests.

## Authors' contributions

WH participated in the design of the study, carried out the synthesis, analyzed the data, and drafted the manuscript. NT participated in the design of the study and helped to draft the manuscript All authors read and approved the final manuscript.
